# Examination Approaches to Brachial Plexus Injury for Neurosurgical Residents

**DOI:** 10.21315/mjms2023.30.6.7

**Published:** 2023-12-19

**Authors:** Umaira Saleh, Guan Yan Tan, Murni Fuad, Jafri Malin Abdullah, Zamzuri Idris, Abdul Rahman Izaini Ghani, Sanihah Abdul Halim

**Affiliations:** 1 Department of Neurosciences, School of Medical Sciences, Universiti Sains Malaysia, Kelantan, Malaysia; 2 Brain and Behaviour Cluster, School of Medical Sciences, Universiti Sains Malaysia, Kelantan, Malaysia; 3 Unit of Neurology, Department of Medicine, School of Medical Sciences, Universiti Sains Malaysia, Kelantan, Malaysia; 4 Department of Neurosurgery, Hospital Pulau Pinang, Pulau Pinang, Malaysia

**Keywords:** brachial plexus, clinical evaluation, neurology, neurosurgery, upper limb

## Abstract

**Background:**

Brachial plexus injury is a severe peripheral nerve injury that affects the upper extremities and causes functional damage and disability. A detailed and accurate clinical examination is required to accurately localise the site of injury. This video manuscript aims to provide guidelines for the structured assessment of a patient with brachial plexus injury, specifically tailored to Malaysian medical students and trainees.

**Methods:**

A video demonstrating the examination of the brachial plexus was made. This video, created at the School of Medical Sciences at Universiti Sains Malaysia (USM), demonstrates the proper examination technique for brachial plexus.

**Conclusion:**

We hope that this video will help students and young doctors evaluate patients with brachial plexus injury and reach accurate localisation of the injury.

## Introduction

The brachial plexus is a complex network of nerves innervating the shoulder girdle and upper extremities (1, 2). It is formed in the posterior cervical triangle by the union of the ventral rami of fifth, sixth, seventh and eighth cervical nerve roots and the first thoracic nerve root (1–4). C4 (prefixed) and T2 (postfixed) rarely co-contribute to this formation, making such occurrence of little clinical significance (1, 2, 4). The brachial plexus can be divided into roots, trunks, divisions and cords (2). The cords then further divided into the major nerve branches of the upper extremities (2).

A detailed examination of the brachial plexus and its terminal branches can be performed within minutes in cooperative patients. This video demonstrates the steps of a comprehensive brachial plexus examination, including inspection, muscle tone, motor and sensory assessment, and reflex assessment. Lesion is then localised using one’s knowledge of peripheral nerve anatomy. The deficit can then be graded for comparison with subsequent assessments.

### General Inspection (5)

Ensure proper exposure of the upper trunk. Examine the shoulder, neck and chest. Look for any bruises, ecchymosis, swelling, laceration or abrasion wounds indicating recent injuries. It is also important to look for any scars that may suggest previous surgical intervention and iatrogenic brachial plexus injury. Check for any torticollis, muscle wasting, and atrophy of the small muscles of the hand.

Asymmetry of the upper limbs, such as the groove sign in deltoid palsy, suggests muscle wasting. Note any characteristic abnormal positions of the upper limbs, such as Erb’s palsy or the waiter’s tip position, Klumpke’s palsy, pointing index finger or ‘Benediction finger,’ claw hand and wrist drop. Look at the patient’s face to assess for Horner syndrome, which may indicate preganglionic pathology. Horner syndrome is characterised by miosis, ptosis, anhidrosis and enophthalmos.

Feel for any bony tenderness and step deformity. Palpate the pulse and compare it to the contralateral limb. A poor pulse volume may suggest subclavian artery injury. Abnormal gait or difficulties with standing or balancing may indicate a spinal cord injury that requires further evaluation.

### Tone

Muscle tone is assessed by feeling the muscle’s resistance to a passive range of motion, which does not require any instrumentation and can be quickly performed (6, 7). Tone is graded on a scale from 0 to 4+ as follows: 0 = no response (flaccidity); 1+ = decreased response (hypotonia); 2+ = normal response; 3+ = exaggerated response (mild to moderate hypertonia) and 4+ = sustained response (severe hypertonia) (8).

The Modified Ashworth Scale is universally accepted and is the current standard for clinical assessment of limb spasticity (6, 7). This scale is used to grade spasticity as follows: 0 = no increase in muscle tone; 1 = slight increase in muscle tone, with a catch and release or minimal resistance at the end of the range of motion when an affected part is moved in flexion or extension; 1+ = slight increase in muscle tone, manifested as a catch, followed by minimal resistance through the remainder (less than half) of the range of motion; 2 = marked increase in muscle tone throughout most of the range of motion, but affected part is still easily moved; 3 = considerable increase in muscle tone, passive movement difficult and 4 = affected part is rigid in flexion or extension (6).

### Motor Examination of the Brachial Plexus (3, 5, 9, 10)

Ask the patient to perform active movements before testing for passive motions. First, examine the normal side to establish a baseline for the normal movement of the joint being tested. When testing a movement, support the limb proximal to the relevant joint, so that the test is limited to the selected muscle group and does not require the patient to fix the limb proximally via muscle contraction. Whenever possible, the action of each muscle should be observed separately, noting its individual power. It has long been customary to use the Medical Research Council (MRC) scale for muscle strength (5). This MRC scale ranges from 0 to 5, according to the maximum force expected for a certain muscle (9, 11), as follows: 0 = no contraction; 1 = flicker or trace contraction; 2 = active movement, with gravity eliminated; 3 = active movement against gravity; 4 = active movement against gravity and resistance and 5 = normal power (9).

However, some of the above grades represent a wide range of function, preventing accurate assessment. A modified MRC grading system can be used, accounting for the evaluation of range of movement (ROM), as follows: 0 = no contraction; 1 = flicker or trace contraction; 2 = active movement, with gravity eliminated; 2–3 = active movement against gravity over less than 50% of the feasible ROM; 3 = active movement against gravity over more than 50% of the feasible ROM; 3–4 = active movement against resistance over less than 50% of the feasible ROM; 4 = active movement against resistance over more than 50% of the feasible ROM; 4–5 = active movement against strong resistance over the feasible ROM, but distinctly weaker than the contralateral side; and 5: normal power (12).

Motor examination of the brachial plexus are listed in the guide (Table 1).

#### The Dorsal Scapular Nerve

This nerve arises directly from the C4 and C5 nerve roots to innervate rhomboideus minor and major muscles. To check for rhomboideus muscle, ask the patient to touch their shoulder blades together. Muscle bellies can be felt and sometimes seen. Alternatively, the patient may press the palm of their hand backward against the examiner’s hand. Weakness causes lateral displacement of the vertebral border of the scapula and of the inferior angle. Atrophy may be obscured by the overlying trapezius muscle.

#### The Long Thoracic Nerve

The long thoracic nerves (C5, C6 and C7) supplies the serratus anterior muscle. The serratus anterior muscle stabilises the scapula against the posterior ribs during arm movement. Hence, any paralysis of the muscle will cause scapular winging. To examine the serratus anterior, have the patient press their hands against a wall.

#### The Suprascapular Nerve

The supraspinatus and infraspinatus muscles are innervated by the suprascapular nerves (C5 and C6), originating from the upper trunk. To test the supraspinatus muscle, ask the patient to abduct the upper arm against resistance. The supraspinatus belly can be seen or felt. With the arm adducted, ask the patient to externally rotate their shoulder against resistance. The examiner’s hand should resist the movement, supporting the elbow and preventing abduction of the arm. The infraspinatus muscle can also be seen or felt.

#### The Axillary Nerve

The axillary nerves (C5 and C6) is a terminal branch of the posterior cord and supply deltoid muscle. To examine the deltoid muscle, the patient abducts the upper arm against resistance. The anterior and middle fibers of the deltoid muscle can then be palpated. To examine for posterior deltoid fibers, ask the patient to retract the abducted upper arm against resistance.

#### The Thoracodorsal Nerve

The thoracodorsal nerves (C6, C7 and C8) supplies the latissimus dorsi muscle. The presence of the latissimus dorsi can be quickly assessed by the cough test. The cough test involves placing both hands under the posterior fold of the arm and asking the patient to cough. There will be involuntary contraction of the latissimus dorsi muscles. To grade the strength, ask the patient to hold their upper arm horizontally and adduct against resistance.

#### The Lower Subscapular Nerve

The lower subscapular nerves (C5, C6 and C7) supply the teres major muscle. To assess the teres major, the patient adducts the elevated upper arm against resistance. The muscle belly of the teres major can be seen and felt. The teres major is typically examined along with the latissimus dorsi muscle (long thoracic nerve).

#### The Lateral and Medial Pectoral Nerve

The pectoralis major muscle has a clavicular head as well as a sternocostal head, which are supplied by the lateral pectoral nerves (C5 and C6) and medial pectoral nerves (C6, C7 and C8). The pectoral muscles are the adductor muscles of the arm. To test the clavicular head of the pectoralis muscles, ask the patient to push forward against the examiner’s hand with upper arm above the horizontal plate. To test for the sternocostal head of the pectoralis muscle, the patient adducts the upper arm against resistance.

#### The Musculocutaneous Nerve

The musculocutaneous nerve (C5 and C6) is a terminal branch of the lateral cord and supplies the biceps brachii muscle. To test the biceps brachii, the patient flexes the supinated forearm against resistance. The muscle belly can be seen and felt. If there is weakness in elbow flexion with the forearm supinated, semi-pronated forearm can still be flexed by the brachioradialis muscle.

#### The Radial Nerve and Posterior Interosseous Nerve

The radial nerves (C5, C6 and C7) is a direct continuation of the posterior cord and supplies the triceps brachii, brachioradialis, supinator, and extensor carpi radialis longus muscles. The posterior interosseous nerves (C7 and C8), which originate from the radial nerve at the radio humeral joint, supply the extensor carpi ulnaris, extensor digitorum, extensor pollicis longus, extensor pollicis brevis and abductor pollicis longus muscles.

To test for triceps brachii, the patient extends the elbow against resistance. The long and lateral heads of the triceps muscle can be seen and felt. To check for brachioradialis, the patient flexes the forearm against resistance, with the forearm midway between pronation and supination. The muscle belly of the brachioradialis can be seen and felt. To check the supinator muscle, the patient supinates the forearm against resistance with the forearm extended at the elbow. To check the extensor carpi radialis longus, the patient extends and abducts the wrist against resistance. To check the extensor carpi ulnaris, the patient extends and adducts the wrist against resistance. The muscle belly or tendon can be felt. For examination of the extensor digitorum muscle, the patient extends the metacarpal phalangeal joint against resistance with the hand supported.

The tendon of the extensor pollicis longus can be seen and felt when the patient extends the thumb at the interphalangeal joint (IPJ) against resistance, whereas the tendon of the extensor pollicis brevis can be seen and felt upon extension of the thumb at the metacarpophalangeal joint (MCPJ) against resistance. The tendon of the abductor pollicis longus can be palpated in close proximity, anterior to the tendon of extensor pollicis brevis, with the patient abducting the thumb at the carpometacarpal joint in a plane perpendicular to the palm.

#### The Median Nerve and Anterior Interosseous Nerve

The median nerve, with root values of C6–T1, supplies the pronator teres, flexor carpi radialis, flexor digitorum superficialis, lumbricals, abductor pollicis brevis and opponens pollicis muscles. The anterior interosseous nerve (C7 and C8) is a terminal branch of the median nerve supplying the flexor pollicis longus, pronator quadratus and lateral half of the flexor digitorum profundus.

To assess the pronator teres muscle, the patient pronates the forearm against resistance. To check for the flexor carpi radialis, the patient flexes and abducts the wrist against resistance. For the assessment of flexor digitorum superficialis muscle, with the proximal phalanx fixed, the patient flexes the finger at the proximal IPJ against resistance. This test does not eliminate the possibility that the flexion of the proximal IPJ is caused by the flexor digitorum profundus. The flexor digitorum profundus muscle is divided into a radial half (supplied by the anterior interosseous nerve) and an ulnar half (supplied by the ulnar nerve). This muscle can be assessed by asking the patient to flex the distal phalanx of the index finger against resistance with the middle phalanx fixed. To check the flexor pollicis longus, the patient flexes the distal phalanx of the thumb against resistance while the proximal phalanx is fixed. The muscle of the abductor pollicis brevis can be seen and felt upon the patient abducting the thumb perpendicular to the palm against resistance. For assess the opponens pollicis, the patient touches the base of the little finger with the thumb against resistance.

Lumbricals 1 and 2 are supplied by the median nerve, and lumbricals 3 and 4 are supplied by the ulnar nerve. The patient extends the fingers at the proximal IPJ against resistance with a hyperextended MCPJ.

#### The Ulnar Nerve

The ulnar nerves (C7, C8 and T1) arise as a continuation of the medial cord of the brachial plexus. It supplies the flexor carpi ulnaris, interossei muscle, flexor digiti minimi, abductor digiti minimi and adductor pollicis muscles. To check the flexor carpi ulnaris, the patient abducts the little finger against resistance. The interossei muscles can be assessed by asking the patient to abduct (dorsal interossei) or adduct the fingers (palmar interossei) against resistance.

To test the flexor digiti minimi, the patient flexes the little finger at the MCPJ against resistance with the finger extended at both IPJs. The muscle belly of the abductor digiti minimi can be felt and seen with the abduction of the little finger against resistance. To check for the adductor pollicis muscle, the patient adducts the thumb at right angles to the palm against resistance.

### Sensory Examination

**Figure 1:**
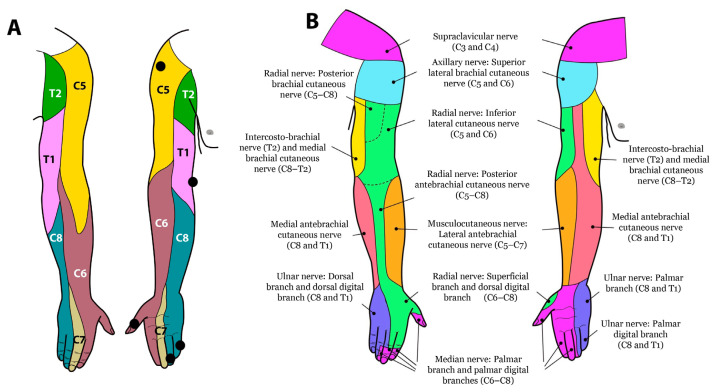
A. Upper limb dermatomal pattern with autonomous zone (dotted); B. Peripheral cutaneous nerves distribution (9)

Sensory examination can be done according to dermatomal pattern or peripheral cutaneous nerve distribution. It is important to distinguish spinal dermatomes and cutaneous nerve distribution in order to localise whether a lesion is affecting the spinal root or a peripheral nerve (5). This is most commonly done in a dermatomal pattern, over a specific ‘autonomous zone’ (5). Autonomous zones are small, distinct areas of skin with minimal to no overlap of sensory innervation by nerve root (5). Assessing the affected limb in these particular zones allows for the most accurate clinical evaluation of sensory function (5). The upper extremity autonomous zones used for evaluation are illustrated in Figure 1. The appreciation of light touch and the pin-prick sensation at each of the key points is separately scored on a three-point scale, the Sensory grading system (ASIA) (5, 11). With comparison to the patient’s cheek as a normal point of reference, sensations are graded as follows: 0 = absent; 1 = altered (impaired or partial appreciation, including hyperesthesia); 2 = normal and NT = not testable (11).

### Reflexes

Reflexes may be the earliest and most subtle changes indicating a disturbance in neuronal function (13). Reflexes are the most objective aspect of the neurologic examination and may be graded as follows: 0 = absent; 1+ or + = sluggish or diminished; 2+ or ++ = normal; 3+ or +++ = exaggerated and 4+ or ++++ = markedly hyperactive (13). The deep tendon reflexes examined in the brachial plexus include the biceps (musculocutaneous nerve C5 and C6), triceps (radial nerve C7 and C8), and brachioradialis (radial nerve C5 and C6) (Table 2) (13).

Inform the patient that the test is complete and thank them for participating. Summarise and report the findings.

### Clinical Utilisation

The challenge of examining a patient with brachial plexus injury lies in the localisation of the level of injury—whether it affects the roots, trunk, cord or distal branches. To determine the level of injury, the following are important brachial plexus nerves and associated muscles examined for evidence of nerve injury (14).

Paralysis and the atrophy of muscles innervated by branches directly off the roots suggest root injury (14). Atrophy of the trapezius and sternocleidomastoid suggests spinal accessory nerve injury rather than brachial plexus injury (5). Other muscles supplied directly from the roots include rhomboideus major and minor (dorsal scapular nerve from C4 and C5) and the serratus anterior muscle (long thoracic nerve from C5, C6 and C7). Injury to C8 and T1 roots that lie in close proximity to the sympathetic ganglion cause Horner syndrome, evident by partial ptosis, miosis, enophthalmos and anhidrosis (4, 14).

The suprascapular nerve arises from the upper trunk (C5 and C6) above the clavicle and innervates the supraspinatus and infraspinatus muscles (3, 9, 14). The nerve to the subclavius cannot be clinically evaluated (14). An upper-trunk injury, Erb’s palsy assumes a characteristic waiter’s tip position due to the unopposed action of the remaining musculature (10). The arm is adducted and internally rotated (unopposed pull of the pectoralis major), the elbow is extended and the forearm is pronated (unopposed pull of the triceps and pronator teres), and the wrist and fingers are flexed (from weak finger and wrist extensors) (10). Injury to the lower trunk entails Klumpke’s palsy, comprised of the C8 and T1 roots, causing significant weakness, atrophy and clawing of the hand (10).

Injury to the lateral cord of the brachial plexus results in weakness of the clavicular head of the pectoralis major muscle due to lateral pectoral nerve palsy (14). The medial cord provides branches to medial pectoral nerve. Hence, examining the pectoral major muscle is necessary to localise a cord-level injury: involvement of the clavicular head of the pectoralis major muscle implies lateral pectoral nerve palsy, while the sternocostal head of the pectoralis major muscle involvement implies palsy of both the lateral and medial pectoralis nerves—that is, both medial and lateral cord injury (14). The posterior cord provides branches to the thoracodorsal and both the upper and lower subscapular nerve, innervating the latissimus dorsi, subscapularis and teres major, respectively.

Patterns of muscular weakness are used to identify injury to one or more nerves, cords or roots. Sensory loss of the upper lateral arm (C5), thumb (C6), long finger (C7), fifth finger (C8) and medial forearm (T1) is then used to confirm the localisation. The following is a step-by-step video of the examination of the brachial plexus.

Video link: https://www.youtube.com/watch?v=X3BQFGLocOg&t=1s

## Conclusion

One’s knowledge of anatomy combined with the correct examination techniques are the keystones of evaluating brachial plexus injuries. We hope that this video presenting a systemic examination of approaches to brachial plexus injuries will help you in obtaining accurate localisation and diagnosis.

## Figures and Tables

**Table 1:** Summary of full motor examination of brachial plexus (9, 10)

Muscle	Segmental innervation	Peripheral nerve	Technique of examination
**Back**			
Rhomboideus major and minor	C4 and C5	Dorsal scapular nerve	Patients press the palm of their hand backward against the examiner’s hand
Serratus anterior	C5, C6 and C7	Long thoracic nerve	Patients press their hands against a wall
Latissimus dorsi	C6, C7 and C8	Thoracodorsal nerve	Hold the upper arm horizontally and adduct against resistance
**Shoulder**			
Supraspinatus	C5 and C6	Suprascapular nerve	Abduct the upper arm against resistance, below 30°
Deltoid	C5 and C6	Axillary nerve	Abduct the upper arm against resistance at below 90°
Infraspinatus	C5 and C6	Suprascapular nerve	With the arm adducted, ask the patient to externally rotate their shoulder against resistance
Teres major	C5, C6 and C7	Lower subscapular nerve	Adduct the elevated upper arm against resistance
Pectoralis major	C5–T1	Lateral pectoral nerve (C5 and C6) Lateral and medial pectoral nerves (C6, C7 and C8)	Clavicular head: Patient to push forward against the examiner’s hand with upper arm above the horizontal plate Sternocostal head: Adducts the upper arm against resistance
**Arm**			
Biceps brachii	C5–C6	Musculocutaneous nerve	The patient flexes the supinated forearm against resistance
Triceps brachii	C6, C7 and C8	Radial nerve	Extends the elbow against resistance
Brachioradialis	C5–C6	Radial nerve	The patient flexes the forearm against resistance, with the forearm midway between pronation and supination
**Forearm**			
Supinator	C6–C7	Radial nerve	The patient supinates the forearm against resistance with the forearm extended at the elbow
Pronator teres	C6–C7	Median nerve	The patient pronates the forearm against resistance
Extensor carpi radialis longus	C5, C6 and C7	Radial nerve	Extends and abducts the wrist against resistance
Extensor carpi ulnaris	C7–C8	Radial nerve (posterior interosseous nerve)	Extends and adducts the wrist against resistance
Flexor carpi radialis	C6–C7	Median nerve	The patient flexes and abducts the wrist against resistance
Flexor carpi ulnaris	C7, C8 and T1	Ulnar nerve	The patient flexes and adducts the hand at the wrist against resistance
**Hand**			
Flexor pollicis longus	C7, C8 and T1	Median nerve (anterior interosseous nerve) C7 and C8	The patient flexes the distal phalanx of the thumb against resistance while the proximal phalanx is fixed
Extensor pollicis longus	C7 and C8	Radial nerve (posterior interosseous nerve)	The patient extends the thumb at the IPJ against resistance
Extensor pollicis brevis	C7 and C8	Radial nerve (posterior interosseous nerve)	The patient extends the thumb at the MCPJ against resistance
Abductor pollicis longus	C7 and C8	Radial nerve (posterior interosseous nerve)	Abduct the thumb at the carpo-metacarpal joint in a plane perpendicular to the palm
Abductor pollicis brevis	C8 and T1	Median nerve	The patient abducts the thumb perpendicular to the palm against resistance
Opponens pollicis	C8 and T1	Median nerve	The patient touches the base of the little finger with the thumb against resistance
Adductor pollicis	C8 and T1	Ulnar nerve	The patient adducts the thumb at right angles to the palm against resistance
Extensor digitorum	C7 and C8	Radial nerve (posterior interosseous nerve)	The patient extends the MCPJ against resistance with the hand supported
Flexor digitorum superficialis	C7, C8 and T1	Median nerve	With the proximal phalanx fixed, the patient flexes the finger at the proximal IPJ against resistance
Flexor digitorum profundus (radial half)	C8 and T1	Median nerve (anterior interosseous nerve) C7 and C8	Flex the distal phalanx of index and middle finger against resistance with the middle phalanx fixed
Flexor digitorum profundus (ulnar half)	C8 and T1	Ulnar nerve	Flex the distal phalanx of ring and little finger against resistance while the middle phalanx is fixed
Lumbricals (radial half and ulnar half)	C8 and T1 C8 and T1	Median nerve (radial half) Ulnar nerve (ulnar half)	Extend the finger at the proximal IPJ against resistance with a hyperextended MCPJ
Interossei (palmar and dorsal)	C8 and T1	Ulnar nerve	Dorsal interosseous muscle: Abduct the index finger against resistance Palmar interosseous muscle: Adduct the index finger against resistance
Flexor digiti minimi	C8 and T1	Ulnar nerve	The patient flexes the little finger at the MCPJ against resistance with the finger extended at both IPJs
Abductor digiti minimi (ADM)	C8 and T1	Ulnar nerve	Abduct the little finger against resistance

**Table 2:** Commonly elicited deep tendon reflexes for brachial plexus (13)

Reflex	Segmental level	Peripheral nerve
Biceps	C5 and C6	Musculocutaneous
Triceps	C7 and C8	Radial
Brachioradialis	C5 and C6	Radial
